# Pleural Effusion/Empyema With Mycobacterium kansasii

**DOI:** 10.7759/cureus.21300

**Published:** 2022-01-16

**Authors:** Kelly F Luttmann, Victoria R Starnes, Kylie Rostad, Katherine K Girdhar, Joan Duggan

**Affiliations:** 1 Infectious Diseases, The University of Toledo, Toledo, USA

**Keywords:** rare cause of pleural effusion, non-tuberculous pleural effusion, non-tuberculous mycobacterium, mycobacterium kansasii pleural effusion, mycobacterium kansasii

## Abstract

*Mycobacterium kansasii* is a nontuberculous mycobacterium that causes pulmonary symptoms, commonly associated with underlying conditions, including malignancy, prior transplant, and HIV. However, rarely does *Mycobacterium kansasii* present with pleural effusion. We present a case of a 56-year-old female who presented with dyspnea and chest pain, and sputum culture was positive for acid-fast bacilli. A CT scan revealed a left-sided pleural effusion. Based on a thorough review of the literature using Embase and PubMed, we found that only 22 cases of a *Mycobacterium kansasii* pleural effusion have been reported. We provide a discussion on maintaining a broad differential in the treatment of immunocompromised individuals with *Mycobacterium* infection.

## Introduction

*Mycobacterium kansasii* is a nontuberculous mycobacterium (NTM) that was first described in 1953 by Buhler and Pollack, who identified this bacterium as a human pathogen in patients with a pulmonary disease resembling tuberculosis [1]. This bacterium is a slow-growing, atypical acid-fast bacillus that is ubiquitously found in the environment, namely tap water [1,2]. Therefore, this pathogen was commonly considered contamination or colonization. We now know that this pathogen can cause a wide range of diseases including lung disease, lymphadenopathy, skin, and soft tissue infection, osteomyelitis, and disseminated disease [2]. These presentations occur more commonly in immunocompromised patients.

*M. kansasii* is the second most common cause of lung disease secondary to an NTM following Mycobacterium avium complex (MAC) in the United States [3,4]. Bronchopulmonary disease is the most frequent presentation of infection with *M. kansasii* and commonly occurs in patients with underlying lung disease [3,5]. Presenting symptoms may include dyspnea, cough, hemoptysis, weight loss, fever, and night sweats [5]. The course of the disease can be indolent, lasting several months to years; however, it may occasionally be rapidly progressive [6,7]. Infection due to *M. kansasii* can be clinically indistinguishable from *Mycobacterium tuberculosis*. Infection is usually diagnosed via bronchoscopy, tissue biopsy, thoracentesis, or pericardiocentesis using culture findings, biochemical testing, or high-performance liquid chromatography. While pleural effusions can be seen with pulmonary disease due to mycobacterium, around 5% of cases are due to* M. tuberculosis*, they are very rarely seen with *M. kansasii* [6].

We present a case of *M. kansasii* pleural effusion and review an additional 22 cases in the English-language literature regarding risk factors for disease, clinical presentation, treatment, and outcome for this unusual infection. This case was previously presented as a meeting abstract at the 2021 Ohio/Air Force Scientific Meeting on October 28, 2021.

## Case presentation

**Figure 1:**
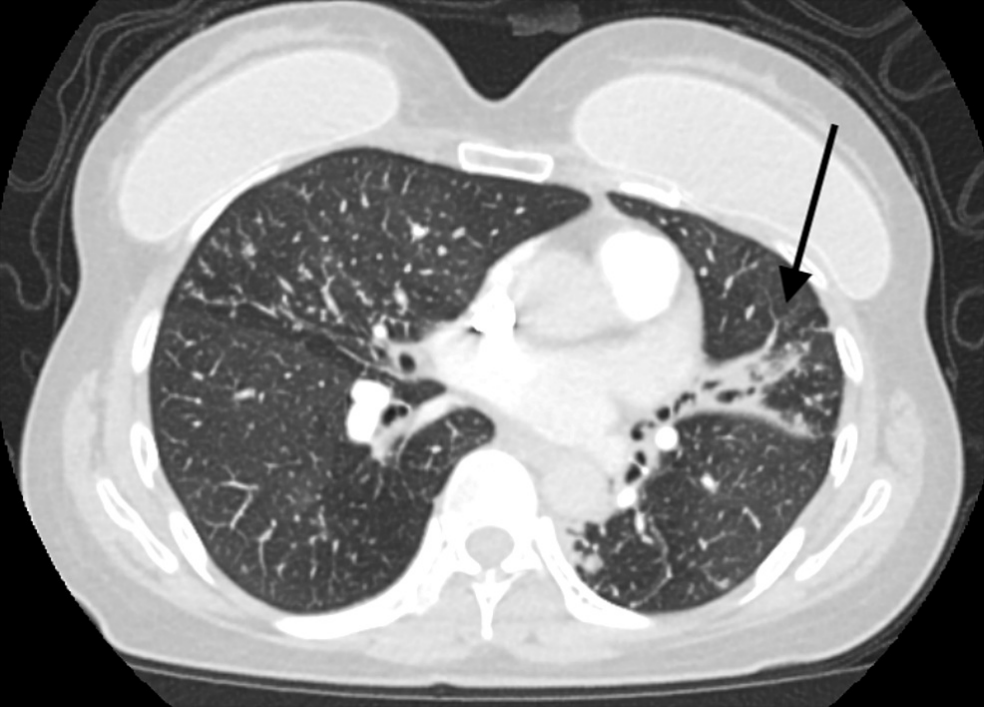
Multifocal nodular opacities in a tree-in-bud distribution

**Figure 2:**
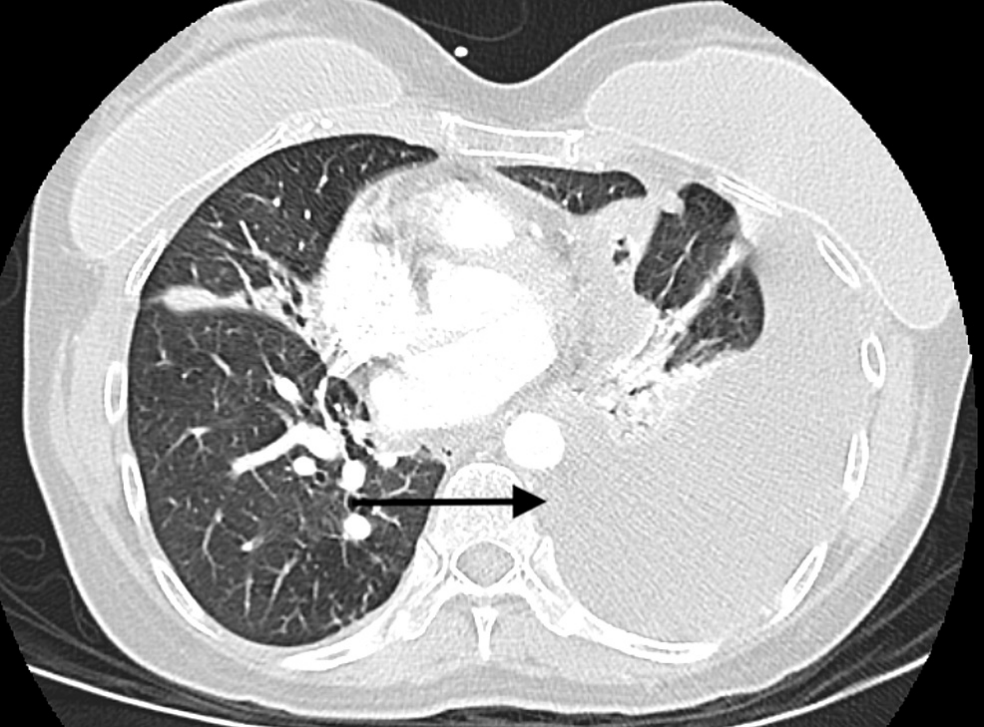
Left pleural effusion at the lung base with tracking along the lateral pleural surface

A 56-year-old female with no significant past medical history presented to the emergency department with shortness of breath and pain in her left chest. Approximately three months prior to her presentation, she was seen in the outpatient setting due to hemoptysis. At that time, she had no associated symptoms and denied fever, chills, night sweats, cough, and nausea but a CT scan of her chest (Figure 1) revealed multifocal nodular opacities in a tree-in-bud distribution, suggestive of atypical mycobacterial or fungal infection. An expectorated sputum sample and follow-up bronchoscopy were positive for acid-fast bacilli and subsequently grew *M. kansasii*. Prior to the start of azithromycin, ethambutol, and rifampin, she presented to the emergency department with shortness of breath and chest pain. A follow-up CT demonstrated a new loculated left pleural effusion at the left lung base with tracking along the left lateral pleural surface (Figure 2). A left-sided chest tube was placed and 1.5 liters of serosanguinous fluid was drained in the first 24 hours. Analysis of the fluid revealed an exudate with LDH of 128U/L, total protein of 5.9g/dL and white blood cell count of 2938 cells/uL with lymphocytic predominance. The fluid was AFB positive on acid-fast smear, however, cultures remained negative. She was ultimately discharged on ethambutol, azithromycin, and rifampin and her clinical condition continues to improve at this time. 

## Discussion

Methods

Case reports of *Mycobacterium kansasii* empyema were identified through a computer-generated search using Embase and PubMed with subsequent review of noted references. Search terms included “mycobacterium kansasii” and “pleural effusion”. Only cases from the English language literature were included. A case was defined by documented *M. kansasii* infection with the recovery of this organism from a culture of either sputum, bronchoscopy, gastric aspirate, or other normally sterile fluid such as blood or cerebrospinal fluid in the presence of empyema without other etiology between 1980 and 2020. Cases of empyema were defined using the standard definition of collection of infected exudate within the pleural cavity. Cases were included in the analysis if enough demographic information was available to allow the identification of individual patients. Demographic information included age and gender. 

Results

Demographics

A total of 22 cases of *M. kansasii* pleural effusion were reported between 1980 and 2020 [2-4,6-21]. These included 13 men (59.0 %) and nine women (40.9%). Ages ranged from 24 to 87 years with a mean age of 52.9 ± 19.5.

Underlying Conditions

Over two-thirds of the patients reviewed were noted to have underlying comorbidities with the majority of these causing a weakened immune system (Table 1). The most common conditions were malignancies and receipt of an organ transplant. Nine patients (39.2%) were taking immunosuppressive medications [4,6,9,12-13,16,18-21].

**Table 1:** Underlying conditions of 23 patients with Mycobacterium kansasii pleural effusion *Some patients had more than one underlying condition † Rheumatoid arthritis (2), pneumothorax (1), prior severe viral infection (1), coronary artery disease (1), COPD(1)

Condition or risk-associated status	No. (%) of patients*
HIV	2 (8.7)
Diabetes mellitus	2 (8.7)
Hypertension	2 (8.7)
Transplant recipient	3 (13.0)
Hematologic malignancy	5 (21.7)
Solid organ malignancy	1 (4.3)
Other †	7 (30.4)
None	6 (26.1)

Pleural Effusion

The laterality of effusion was identified in 14 cases (60.9%). Seven (50%) of these patients had a right-sided effusion [4,7,9-11], three (21.4%), including our case, had a left-sided effusion [13] and four (28.9%) had bilateral effusions [8,13-14,21]. Two left pleural effusions were reported as large [4,8] and two right pleural effusions were reported as small [7,11]. Otherwise, size was not reported. The pleural effusions were found to be exudative in five (21.7%) cases [6-8,14]; the other 18 cases did not report pleural fluid studies to be able to determine this information. Of the cases, 15 were isolated pleural effusions, while eight showed pulmonary signs of pneumothorax [7,8], cavitation [4,12], abscess [4], infiltrate [10,13,14], and lung nodules [15].

Diagnosis of pleural effusion involved computed tomography (CT) scan in 12 (52.2%) cases [7-11,13-15,18,20-21], and chest radiograph in nine (39.1%) [5,7-9,11,12,14,18,20]. Seven cases had both imaging studies performed [7-9,11,14,18,20].

Cultures of the pleural fluid were reported in all cases. All cases were noted to have cultures positive for *M. kansasii* except for one where cultures were negative. In this case, the patient was noted to have a positive culture previously [14].

Symptoms and Signs

Fifteen (65.2%) of the cases described reported signs and symptoms of the patient upon presentation and throughout the hospital stay (Table 2). The most commonly reported symptoms included fever, pulmonary symptoms including chest pain, cough, and dyspnea as well as constitutional symptoms. Cases with suspicion of disseminated disease presented atypically. The primary complaints of these patients included gastrointestinal and cardiac symptoms [4,12,15].

**Table 2:** Signs and symptoms of 15 patients with Mycobacterium kansasii pleural effusion

Symptom	Number of Patients (%)
Fever	11 (73.3)
Chills	2 (13.3)
Constitutional symptoms (weight loss, headache, fatigue, night sweats, and malaise)	7 (46.7)
Pulmonary symptoms	13 (86.7)
Chest pain	6 (40.0)
Dry cough	5 (33.3)
Productive cough	1 (6.7)
Hemoptysis	1 (6.7)
Dyspnea	7 (46.7)
Gastrointestinal symptoms	1 (6.7)
Cardiac symptoms (cardiopulmonary arrest, QT prolongation, asystole)	2 (13.3)

Treatment

Definitive therapy was reported for 19 of the 23 cases (82.6%). Most patients initially received some variation of RIPE therapy consisting of rifampin (RFP) and isoniazid (INH) with either pyrazinamide (PZA) or ethambutol (EB) or, in some cases, both PZA and EB (Table 3). In all the cases that mentioned therapy, the patients were found to have received combination therapy. The most common combination included RFP, INH, and EB only. Of the cases reporting the use of RIPE, only one mentioned additional therapy with prednisone; it is noted that this patient did survive [14]. One case reported the use of open-window thoracostomy in management [9]. The duration of treatment was reported for only six patients: two for ≤6 months, two between 6-12 months, and two for >12 months. Susceptibility of the bacterial isolates was reported in three cases [10,14-15].

**Table 3:** Medication regiment and outcome of 19 patients with Mycobacterium kansasii pleural effusion *Reported based on final treatment (before discharge/death) RFP: rifampin; INH: isoniazid; PZA: pyrazinamide; EB: ethambutol

Medication Regiment*	Number of Patients (%)	Outcomes (%)
INH, RFP, EB	11 (57.9)	Survived: 8 (72.7)
		Died: 2 (18.2)
		Unknown: 1 (9.1)
INH, RFP, EB, PZA (RIPE therapy)	3 (15.8)	Survived: 3 (100)
		Died: 0
		Unknown: 0
EB, RFP + another class	3 (15.8)	Survived: 3 (100)
		Died: 0
		Unknown: 0
Ofloxacin, Clofazimin, Azithromycin	1 (5.3)	Survived: 0
		Died: 0
		Unknown: 1 (100)

*Complications and Outcomes*.

The outcome was reported for 21 of the 23 cases (91.3%), with 14 surviving, five dying, and two being lost to follow-up. While the causes of death varied (Table 4), the most commonly reported was overwhelming sepsis while receiving treatment in the inpatient setting [13,15].

**Table 4:** Cause of death in five patients with Mycobacterium kansasii pleural effusion DIC: disseminated intravascular coagulation

Cause of death	No. (%) of patients
Sepsis	2 (40%)
Cardiopulmonary arrest	1 (20%)
DIC/multi-organ failure	1 (20%)
Unspecified	1 (20%)

Discussion

*Mycobacterium kansasii* is an NTM known to be a ubiquitous environmental pathogen, found mainly in tap water. It is the second most common NTM in AIDS patients, after MAC [4]. There are seven identified genotypes of *M. kansasii* with Types I and II being the most common clinical isolates. Type I is most likely responsible for infections in Europe, the United States, and Japan [22]. Infection usually occurs via aspiration or local environmental inoculation via the aerosol route and there is little evidence of person-to-person transmission.

The incidence of *M. kansasii* infection increased following the emergence of HIV. Longitudinal prevalence studies have been inconsistent, which is thought to be related to considerable regional variability [22]. In a survey prior to the HIV epidemic, the annual incidence was 0.5 per 100,000 in 44 states of the United States. However, the incidence of infection is estimated to be as high as 532 cases per 100,000 in populations with HIV [22]. Rates are relatively high in England, Wales, and South America with increasing incidence in Israel, Korea, Spain, Portugal, France, Brazil, and Japan.

In HIV-positive patients with advanced immunosuppression, the lung is the most common organ involved in *M. kansasii* infection [23,24]. It is reported that 20% of HIV-positive patients with *M. kansasii* infection will develop disseminated disease [23]. Of the two cases with HIV-positive individuals in our review, neither developed disseminated disease and both survived [10,11]. This review demonstrated fewer cases in patients who had HIV as an underlying condition than initially anticipated considering widely supported data about differential rates of transmission. Despite being statistically insignificant, it is noteworthy that four cases reported hematologic malignancy, including myelodysplastic syndrome, hairy cell leukemia, chronic myelogenous leukemia, and lung cancer.

The most common causes of pleural effusion are congestive heart failure, cancer, bacterial pneumonia, and pulmonary embolism [25]. Pleural effusions secondary to bacterial pneumonia are characterized by exudate with associated symptoms of cough, fever, and infiltrate. Signs and symptoms were not reported for all cases, and the effusions in only five cases were distinguished as exudate. Symptoms typically seen in pulmonary *M. kansasii* include cough, sputum production, weight loss, shortness of breath, hemoptysis, and fever and sweats [23]

Five of the cases presented patients who died during their hospital course. One case did not report the reason for death, but in case of the other four patients, deaths occurred in the hospital, with conditions not directly related to the *M. kansasii* pleural effusion (cardiopulmonary arrest, sepsis, multi-organ failure).

Despite the 22 cases reported in the literature, data was not consistently reported across the case reports. Without consistent data, large conclusions cannot be drawn. Reporting guidelines, such as the Case Reports (CARE) guidelines, should be implemented to ensure transparency and complete reporting [26]. The CARE guidelines include demographic information, main symptoms, medical history, physical exam, intervention, outcomes, and other items to be included in a case report. The inclusion of many of these items consistently across reviewed cases may have improved the conclusions of this paper.

## Conclusions

While our patient presented without the symptoms typically associated with *Mycobacterium* infections, including fever, chills, night sweats, and cough, a CT scan was indicative of atypical mycobacterial infection. Follow-up sputum sample and bronchoscopy guided the diagnosis of *M. kansasii* infection, rather than the more common *M. tuberculosis*. Through this case report, we hope to encourage others to keep a broad differential, especially in immunocompromised patient populations, as the treatments differ.
